# Comparison of heat and mass transfer of different microwave‐assisted extraction methods of essential oil from *Citrus limon* (Lisbon variety) peel

**DOI:** 10.1002/fsn3.240

**Published:** 2015-05-05

**Authors:** Mohammad‐Taghi Golmakani, Mahsa Moayyedi

**Affiliations:** ^1^ Department of Food Science and TechnologySchool of AgricultureShiraz UniversityShirazIran; ^2^ Department of Food Science and TechnologySchool of Agriculture4‐Varamin Branch, Islamic Azad University VaraminIran

**Keywords:** Antioxidant, *Citrus limon*, essential oil, extraction, microwave

## Abstract

Dried and fresh peels of *Citrus limon* were subjected to microwave‐assisted hydrodistillation (MAHD) and solvent‐free microwave extraction (SFME), respectively. A comparison was made between MAHD and SFME with the conventional hydrodistillation (HD) method in terms of extraction kinetic, chemical composition, and antioxidant activity. Higher yield results from higher extraction rates by microwaves and could be due to a synergy of two transfer phenomena: mass and heat acting in the same way. Gas chromatography/mass spectrometry (GC/MS) analysis did not indicate any noticeable differences between the constituents of essential oils obtained by MAHD and SFME, in comparison with HD. Antioxidant analysis of the extracted essential oils indicated that microwave irradiation did not have adverse effects on the radical scavenging activity of the extracted essential oils. The results of this study suggest that MAHD and SFME can be termed as green technologies because of their less energy requirements per ml of essential oil extraction.

## Introduction

Citrus is a genus of the Rutaceae family, and it is one of the most common tree fruit crops in the world, with an annual production of approximately 123 million tons in 2010. Citrus is a genus comprised of several important species, the most relevant of which is orange. On a worldwide basis, oranges constitute 56% of the total Citrus crops. Tangerines, mandarins, and clementines make up 17%. Lemons (*Citrus limon*) and limes comprise 11%, whereas grapefruit (with pomelo) comprises only 6% of the total Citrus crops produced worldwide (Abbate et al. 2012). *C. limon* is the third most important cultivated citrus species, after orange and mandarin, with a global production of 4,200,000 metric tons each year (Boluda‐Aguilar and López‐Gómez 2013). Among *C. limon* cultivars, “Lisbon” is a major variety (Ladaniya 2008).

Today's health care issues and its pertinent concerns have driven man to utilize natural substances in a wide array of industrial fields. Essential Oils (EOs) are one of the most renown of those natural substances. There are hundreds of EOs and complex aromatic substances which are widely used in foods, drugs, and cosmetics (Luque de Castro et al. 1999). The main advantage of EOs is that they are generally recognized as safe (GRAS) and can be used in any foods, as long as their maximum effects are attained with the minimum change to the organoleptic properties of the food (Viuda‐Martos et al. 2008). EOs obtained from *C. limon* have been industrially applied in many foods and beverages so far. Each lemon fruit gives 30–40% peel with respect to the whole fruit. Since *C. limon* EOs are mainly located in the fruit peel, their extraction is economically affordable, because the fruit peel is considered as a waste for the fruit juice industry (Lucker et al. 2002; Settanni et al. 2012).

Cold‐pressed EOs from the peels are the first by‐products to be obtained during the processing of citrus fruits and any improvement in this process would be of great interest for the citrus processing industry (Coll et al. 1995). Another classical production method of citrus EO is hydrodistillation (HD) which yields a volatile oil from citrus, mainly consisting of monoterpenes and a few sesquiterpenes plus oxygenated derivatives (alcohols, aldehydes, ketones, acids, phenols, lactones, acetals, ethers, and esters) (Luque de Castro et al. 1999; Pourmortazavi and Hajimirsadeghi 2007). However, this method is accompanied by several disadvantages such as losses in the volatile compounds, longer extraction times, degradation of some components through thermal and hydrolytic effects, and the questionable quality of the final product (Bayramoglu et al. 2008; Rezvanpanah et al. 2008). Therefore, in the last few years there has been an increasing demand for novel process technologies. Extraction is a new concept to meet the challenges of the 21st century, to protect both the environment and consumers, and in the meantime enhance competition of industries to be more ecologic, economic, and innovative (Chemat et al. 2012). Pioneering ideas led to the understanding that pollution and hazards have to be eliminated right off when released from the pollution source, thus reducing environmental impacts and costs. Novel microwave‐assisted extraction (MAE) (Kaufmann and Christen 2002) methods such as microwave‐assisted hydrodistillation (MAHD) (Golmakani and Rezaei 2008a,b; Wang et al. 2010) and solvent‐free microwave extraction (SFME) (Filly et al. 2014) have proven to be fast and efficient methods for extracting EOs from medicinal plants.

Microwave‐assisted hydrodistillation is an advanced HD method which makes use of a microwave oven. The efficiency of MAHD strongly depends on the dielectric constant value of water and the matrix (Rezvanpanah et al. 2008). MAHD has been used for extracting EOs from *Zataria multiflora* Boiss. (Golmakani and Rezaei 2008b) and Mango (*Mangifera indica* L.) flower (Wang et al. 2010). Solvent‐free microwave extraction is based on the combination of microwave heating and dry distillation. In contrast to MAHD, SFME does not use a large quantity of water (Lucchesi et al. 2004). Solvent‐free microwave extraction has been employed to obtain EOs from ajowan, cumin, and star anise (Lucchesi et al. 2004). Other plant materials that have had their EOs extracted via SFME are cardamom seeds (Lucchesi et al. 2007) and laurel (Bayramoglu et al. 2009; Uysal et al. 2010). MAE (which includes MAHD and SFME) is now recognized as an efficient technique that can reduce the extraction time dramatically. It can increase the yield and the quality of EOs, reduce solvent consumption, lessen pollution, and reduce sample preparation costs (Farhat et al. 2011).

Even though many studies have reported the extraction of EOs from *C. limon*, none of them are based on the use of microwave energy for the extraction of EOs from fresh and dried specimens. Therefore, the aim of this work was to use the MAHD and SFME techniques for the extraction of EOs from dried and fresh *C. limon* peels, respectively. Another attempt was to compare their extraction time, extraction yield, physical constants, chemical compositions, antioxidant activities, and energy consumptions with those of the conventional HD method.

## Materials and Methods

### Chemicals

The 2,2‐diphenyl‐1‐picrylhydrazyl (DPPH) free radical, BHT (butylated hydroxytoluene), vitamin C (L‐ascorbic acid), methanol, and hexane were supplied by Sigma‐Aldrich (St. Louis, MO).

### Plant materials

The lemons used in this study were *C. limon* which were gathered in March 2012 from an experimental plantation located in the Jahrom region, Fars province, Southern Iran (28°50' N latitude, 53°56' E longitude). The genus and cultivar of the experiment's *C. limon* were further approved by a plant taxonomy expert in the Department of Horticultural Science, College of Agriculture, Shiraz University, Shiraz, Iran. The “Lisbon” cultivar of *C. limon* was peeled by hand so as to separate the external part of the *C. limon* (flavedo), rearing a peel yield of 34.58 ± 3.14% (w/w) with respect to the whole fruit. The *C. limon* peel was then placed on a large screen tray for 3 days and was left to dry under ambient conditions (30–40°C). The dried peels were then packed in polyethylene (PE) bags and kept in a dark and cool place until extraction was due. Moisture contents of fresh and dry *C. limon* peels were measured in triplicates according to AACC (1983) method 44–19, using a laboratory oven at 105°C until constant weight was achieved. The moisture contents of fresh and dry peels were 84.78 ± 0.79 and 5.33 ± 0.96% (w/w), respectively. All values are reported on a moisture‐free basis.

### Comparison of different extraction methods

#### Microwave‐assisted hydrodistillation

In employing MAHD, we used a domestic microwave oven (ME3410W, Samsung Malaysia Electronics, Kuala Lumpur, Malaysia) with a wave frequency of 2450 MHz. The input power consumption of microwave oven was monitored using a separate Wattmeter at the entrance of the electrical power supply and the energy consumption was monitored using the designed software. The average input power of microwave oven was 1200 W. The dimensions of the PTFE‐coated cavity of the microwave oven were 23.9 × 37.5 × 38.6 cm. The microwave oven was modified by drilling a hole at the top. A flask with a capacity of 1000 mL was placed inside the oven and was connected to the Clevenger‐type apparatus through the hole. Then, the hole was closed with PTFE to prevent any loss of the heat inside.

Fifty grams of dried *C. limon* peel and 450 mL of distilled water (peel‐to‐water ratio of 1:9) were placed in the reactor and heated by microwave irradiation with 1200 W (100% power) for 15 min. The different densities and their immiscibility required that the water and EO be separated from each other and the excess water be refluxed to the extraction vessel (Rezvanpanah et al. 2008) in order to provide uniform conditions of solid‐to‐liquid ratios for extraction. Every 5 min (i.e., 5, 10, and 15 min), the collected EO was decanted from the condensate. Yields are reported in grams of EOs per 100 g of dried *C. limon* peel.

#### Solvent‐free microwave extraction

Solvent‐free microwave extraction was employed via a similar oven as the one described for MAHD. The SFME is based on the combination of microwave heating and distillation without any water being added. In this method, fresh *C. limon* peels were placed in the microwave oven. The internal heating of the in situ water within the *C. limon* peel distends the EO glands and makes them burst, thus freeing the EOs, which are subsequently evaporated along with the in situ water content of *C. limon* peels. The EO then passes through a condenser outside the microwave cavity where it is condensed. The distillate is collected continuously and forms a two‐phased liquid content (i.e., EO and in situ water) (Uysal et al. 2010).

As for SFME, fresh *C. limon* peels and distilled water, which were of a peel‐to‐water ratio of 1:1, were placed in the microwave reactor using a fixed power of 1200 W. Even though SFME is considered to be a solvent‐free extraction method, we used an equal ratio of water to fresh *C. limon* peels in order to prevent the peels from burning and to ensure that sufficient amounts of water would subsist during the refluxing. The extraction time was fixed at 15 min until no more EO could be obtained further. During this period, the gathered EO was decanted from the condensate in 5 min intervals (i.e., 5, 10, and 15 min).

#### Hydrodistillation

Hydrodistillation was employed more or less like MAHD, but an Electromantle heater (EM2000/C, 335 W, Electrothermal Engineering Ltd., Rochford, UK) was used instead of the microwave oven. *C. limon* peels and distilled water were placed into the HD with a Clevenger‐type apparatus, and EOs were extracted for 120 min, in 30 min intervals (i.e., 30, 60, 90, and 120 min). The EOs obtained from the three extraction methods were collected, dried with anhydrous sodium sulfate, and stored in amber vials at 4°C until further analysis.

#### CO_2_ Emission

The measurements of CO_2_ emitted were carried out based on the procedures mentioned in the previous studies: to obtain 1 kWh of energy from coal or fossil fuels, 800 g of CO_2_ will be released into the atmosphere during their combustion (Ferhat et al. 2006).

### Physical constants

The usual physical constants (specific gravity, refractive index, color, and visual appearance) of the EOs from the *C. limon* peels extracted by the three extraction methods (HD, MAHD, and SFME) were analyzed according to the methods outlined by the Food Chemical Codex (FCC) (Burdock 2010). The specific gravity and the refractive index were measured at 25 and 20°C, respectively. Color of the EOs was defined by a number of parameters: L* (lightness), a* (redness‐greenness), and b* (blueness‐yellowness). These were determined according to the method described by Afshari‐Jouybari and Farahnaky (2011). In the L*a*b* color space, color difference can be expressed as a single numerical value, ΔE*_ab_, which indicates the size of the color difference but not in what way the colors are different. ΔE*_ab_ of the EOs was calculated according to the following equation (Nielsen 2010):

(1) $$\Delta E{\text{*}}_{\mathrm{ab}}=\sqrt{{\left(\Delta L\text{*}\right)}^{2}+{\left(\Delta a\text{*}\right)}^{2}+{\left(\Delta b\text{*}\right)}^{2}}$$

### Scanning electron microscopy

In order to elucidate each MAE procedure and to understand the extraction mechanism, samples were ready to be scanned by electron microscopy when the extraction procedure had finished. For the MAHD and SFME, the scanning was done after a time of 15 min had elapsed from the start of the extraction process. The *C. limon* peels were freeze‐dried using a freeze drier (Armfield, UK) and fixed on the specimen holder with aluminum tape and then sputtered with gold in a Polaron SC 7640 Sputter Coater (Quorum Technologies Ltd., Newhaven, UK), All the specimens were examined with a SEM (Cambridge, UK) under high‐vacuum conditions at an accelerating voltage of 20.0 kV and a working distance of 15 mm (i.e., the distance between the surface of the sample and the microscope lens).

### Gas chromatography/mass spectrometry (GC/MS) identification

The EO constituents obtained via the different extraction methods (HD, MAHD, and SFME) were identified by a gas chromatography (7890A, Agilent Technologies, Santa Clara, CA) coupled with a mass spectrometer (5975C, Agilent Technologies, Santa Clara, CA) operating at 70 eV ionization energy, 0.5 s/scan, and a mass range of 35–400 atomic mass unit (amu), equipped with a HP‐5MS capillary column (5% Phenyl Polysilphenylene‐siloxane; 30 m length; 0.25 mm internal diameter; 0.25 μm film thickness). The injector and detector temperatures were maintained at 280°C. The temperature timeline of the oven was programmed as follows: the initial temperature was set to be 60°C and was then allowed to increase to 210°C (at a rate of 3°C/min). Finally, the temperature increased to 240°C (at a rate of 20°C/min) whereupon 240°C was held constant for 8.5 min. One microlitre of the sample was injected into the GC/MS with the injector being in the split mode (split ratio of 1/100). Helium was used as the carrier gas with a flow rate of 0.9 mL/min. Data in relative percentages were obtained via the electronic integration of peak areas without the use of correction factors. Then, the MSD ChemStation Software (G1701EA, E.02.01.1177, Agilent Technologies, Santa Clara, CA) was used to handle mass spectra and chromatograms.

Most constituents were tentatively identified by comparison of their retention indices (RIs), established in accordance with reference to an homologous series of C_5_–C_28_ n‐alkanes that had been injected following the injection of the EOs under the same chromatographic conditions. Identifications were confirmed when possible by comparisons of their mass spectral fragmentation patterns with those stored in the database bank (Wiley libraries/NBS) and with the mass spectra of relevant literature data (Adams 1995).

### Antioxidant activity: DPPH radical scavenging activity

The antioxidant activities of the EOs from *C. limon* peels were measured by considering the hydrogen donating molecules or the EO's radical scavenging ability, using the stable DPPH free radical (Brand‐Williams et al. 1995). The effect of an antioxidant on DPPH radical manifests itself in the antioxidant's hydrogen donating ability or radical scavenging activity. Mixing the DPPH radical solution with a substrate that donates hydrogen atoms can turn DPPH radical into its reduced (nonradical) form and causes the simultaneous transformation of its color from violet to pale yellow. DPPH radical scavenging activity is shown by the IC_50_ value, defined as the concentration of the antioxidant required for the DPPH radical activity to diminish by 50% (Mazidi et al. 2012). Four ml of various concentrations of the EOs (2, 4, 6, 10, and 50 mg/mL), dissolved in methanol, were added to 2 mL of the 0.2 mmol/L methanolic solution of DPPH radical. The mixtures were shaken vigorously and were then left in the dark at room temperature for 60 min. The absorbance was measured at 517 nm against the blank (methanol). The DPPH radical discoloration of the samples was calculated in percentages according to the following equation (Kulisic et al. 2004):

(2) $$\text{Inhibition}\%\;=100\times \left(A_{\mathrm{Blank}}-A_{\mathrm{Sample}}\right)/A_{\mathrm{Blank}}$$

The EO concentration that provides 50% inhibition (IC_50_) was determined through the inhibition percentages plotted against EO concentrations. All the determinations were carried out in triplicates. BHT and vitamin C were used as a positive control against antioxidant activity.

### Statistical analysis

Statistical analyses of data (differences among HD, MAHD, and SFME) were determined via SAS (Statistical Analysis Software, Version 9.1; SAS Institute Inc., Cary, NC). Evaluations of chemical and physical constants of the extracted EOs were done in triplicates. A general linear model (GLM) procedure was performed to determine significant differences among the three extraction methods (*P *<* *0.05).

## Results and Discussion

### Temperature profile

**Figure 1:**
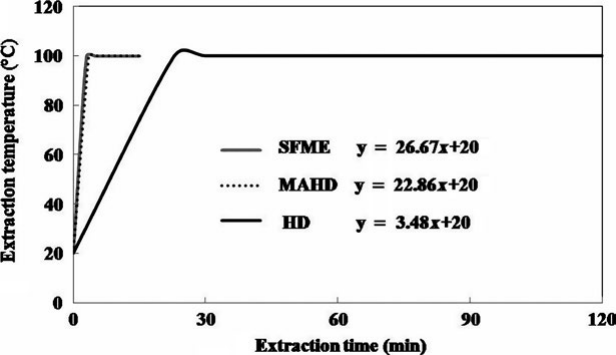
Time‐temperature profile of *Citrus limon* peel essential oil extraction with hydrodistillation (HD), microwave‐assisted hydrodistillation (MAHD), and solvent‐free microwave extraction (SFME) methods.

Figure 1 shows the temperature profile during extractions by HD, MAHD, and SFME from EOs of *C. limon* peels. In all extraction methods, the initial temperature of samples was 20°C. The extraction temperature was equal to the boiling point of water (100°C) at atmospheric pressure as regards HD, MAHD, and SFME. The first EO droplets were observed after 23.0 min in HD, 3.5 min in MAHD, and 3.0 min in SFME. The most important reason for this difference is that MAHD and SFME apply three ways of heat transfer within the samples, namely irradiation, conduction, and convection. On the other hand, heat transfer in HD takes place through conduction and convection only (Golmakani and Rezaei 2008a,b). The rapid temperature rise in MAHD and SFME is the reason behind the time reduction accordingly.

The rate of temperature rise was measured by determining the slope of the linear part of the temperature profile (Fig. 1). The details in Table 1 show that the rates of temperature elevations in the MAHD and SFME methods were greater by sevenfold compared to that of the HD method. This phenomenon can also be attributed to the high dielectric constant of water which absorbs the irradiation from the microwaves and causes a more rapid rise in temperature, compared to the case of HD (Kaufmann et al. 2001). These results are in good agreement with the findings of Mazidi et al. (2012). They found that in comparison to the conventional HD, the MAHD method can accelerate the rate of extraction by increasing the temperature rapidly and by causing the quicker rupturing of EO glands in Black Zira. Also, Ferhat et al. (2006) showed that heating the samples for 3 min was enough for the SFME to reach the extraction temperature (100°C) and to obtain the first EO droplets from orange peels. This is in comparison with the 30 min required by the HD.

**Table 1:** The effect of hydrodistillation (HD), microwave‐assisted hydrodistillation (MAHD), and solvent‐free microwave extraction (SFME) of *Citrus limon* essential oil (EO) on the extraction kinetics

Extraction parameter	HD	MAHD	SFME
Rate of temperature increase (°C/min)	3.48^c^ * ± 0.27	22.86^b^ ± 1.75	26.67^a^ ± 2.04
Starting time of EO accumulation (min)	23.00^a^	3.50^b^	3.00^c^
Total extraction time (min)	120.00^a^	15.00^b^	15.00^b^
Extraction duration (min)	97.00^a^	11.50^c^	12.00^b^
Yield (%, w/w)	1.22^a^ ± 0.14	1.18^a^ ± 0.08	1.36^a^ ± 0.06
Rate of EO accumulation (g/min)	0.01^c^ ± 0.00	0.08^b^ ± 0.00	0.09^a^ ± 0.00

*In each row means with different letters are significantly different (*P *<* *0.05).

### Extraction kinetics

Table 1 shows the effect of different extraction methods on total extraction time, extraction duration (i.e., the difference between the total extraction time and the time when the first droplets of EOs begin to appear), yield and rate of EO accumulation. MAHD and SFME were clearly quicker than the conventional HD. Full recovery of EOs was achieved within the first 15 min of operation in MAHD and SFME, whereas it took at least 120 min for the HD to fulfill the extraction operation. Farhat et al. (2011) found that less time was needed for EOs to be extracted thoroughly from orange peels via microwave extraction (12 min) than via the HD (40 min). Also, Bousbia et al. (2009) presented similar findings in their study on EOs extracted from lime wherein it took 15 min for the microwave hydrodiffusion and gravity method to accomplish the extraction process, in comparison with HD which took 180 min.

As is shown in Table 1, there were no significant differences in the final yields obtained by HD (1.22 ± 0.14% w/w) after 120 min, MAHD (1.18 ± 0.08% w/w) and SFME (1.36 ± 0.06% w/w) after 15 min. Since the EO quantity of the samples was constant and since we had no EO loss caused by evaporation, the final yields in all the three extraction methods (HD, MAHD, and SFME) were equal. Bayramoglu et al. (2009) extracted EO from Laurel and reported that there were no significant differences in the maximum EO yields extracted by HD and SFME. Furthermore, Golmakani and Rezaei (2008a) investigated the effect of different extraction methods on EO yields of *Zataria multiflora* Boiss. They found that the final yields in HD and MAHD were 3.44 and 3.66%, respectively, and that there were no significant differences among the yields obtained through HD and MAHD.

The rates of EO accumulation observed in HD, MAHD, and SFME are shown in Table 1. This index was obtained by dividing the amount of extracted EOs (g) by the corresponding total extraction time (min), which equaled the average rates of EO accumulation (g/min). The results show that the average rates of EO accumulation by MAHD and SFME were at least eight times greater than that of HD. Additionally, the findings revealed that the shorter extraction time in MAHD and SFME was not only caused by their earlier onset of extraction, but also by their higher extraction rates which is mainly due to the more efficient heat transfer conducted by the microwave (Golmakani and Rezaei 2008a).

**Figure 2:**
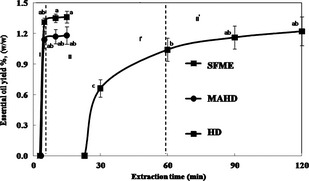
Extraction yield as a function of time in hydrodistillation (HD), microwave‐assisted hydrodistillation (MAHD), and solvent‐free microwave extraction (SFME) of essential oil from *Citrus limon* peel.

According to Figure 2, the extraction yields by MAHD and SFME were 1.14 ± 0.08 and 1.31 ± 0.04% w/w, respectively, after 5.0 min. These yields were significantly similar to the amount of yield obtained by the traditional HD after 60.0 min. Therefore, the extraction time in HD was 12 times lengthier than that of the MAE (MAHD and SFME). Microwave extraction offers a rapid delivery of energy to a total volume of water and also to the *C. limon* peel matrix with a subsequent heating of the water and the *C. limon* peel matrix. This delivery of energy via the microwave occurs efficiently and homogeneously. Since the water within the *C. limon* peel matrix absorbs microwave energy, cells are ruptured by internal superheating which facilitates diffusion of chemicals from the matrix, thus improving the recovery of EO.

**Figure 3:**
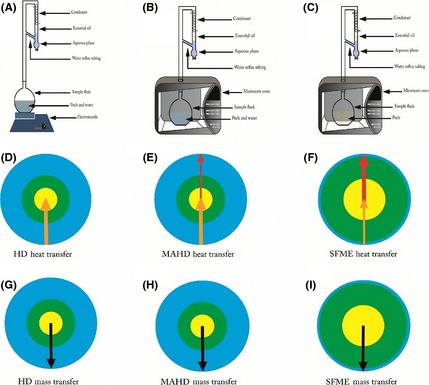
A schematic display of heat and mass transfer during hydrodistillation (HD), microwave‐assisted hydrodistillation (MAHD), and solvent‐free microwave extraction (SFME) of essential oil from *Citrus limon* peel.

As it is shown in Figure 2, the extraction patterns for the three methods were similar, and two phases were observed in the process of extraction kinetics. The first part (I and I') was presented by an ascending line, which denotes the rapid increase in the yield and which represents approximately 85.24, 96.61, and 96.32% of the total yield in HD, MAHD, and SFME, respectively. In the second part, II and II' correspond to a horizontal line which marks the end of the extraction process. The rapid increase in the yield during the first step suggested that the EO was easily accessible by the steam. Indeed, the microwave irradiations distended the *C. limon* peel and lead to the rupture of the glands. However, one of the most striking differences observed between the MAE (MAHD and SFME) and HD methods is the ability of the MAE process to raise the extraction yield of the sample quickly and notably, within a short time. This higher rate of yield is a result of the higher extraction potential of microwaves and could be due to a synergy combination of the two transfer phenomena – mass and heat – acting in the same way. This could be explained by the fact that the mass transfer occurs from the inside to outside in the HD, MAHD, and SFME methods (Fig. 3G–I). The rate of heat transfer differed among the MAE (MAHD and SFME) and the HD. In the case of the HD, heat transfer occurred from the outside to the inside, exclusively because of conduction and convection happening through the water surrounding the *C. limon* peels (Fig. 3D). However, we can suggest that the extraction mechanism of EO obtained by MAHD is partly due to internal heating of in situ water under microwaves irradiation from the inside to the outside of *C. limon* peels, and also mostly due to heat transfer from the outside to inside, similar to the case of HD (Fig. 3E). In SFME, heat transfer partly occurred from the outside to the inside and mostly from the inside to the outside of the *C. limon* peel, which facilitates oil diffusion from the inside of the peel via steam by an increase in the extraction yield due to the synergy combination of the two transfer phenomena – mass and heat – acting in the same direction (i.e., from the inside to the outside) (Fig. 3F). SFME resulted in significant internal heating, thus creating significantly higher internal pressures which promote the bursting of glands and EO extraction from *C. limon* peel (Bayramoglu et al. 2008). This is in agreement with the findings of Farhat et al. (2010) who extracted EOs from caraway seeds using microwave dry‐diffusion and gravity. They reported that the extraction mechanism of EO by microwave dry‐diffusion and gravity is partly due to the internal heating of EO molecules under microwave irradiation from the inside to the outside of caraway seeds without the condition of in situ water and also partly due to a synergy combination of the two transfer phenomena – mass and heat – acting in the same direction.

### Structural changes after extraction

**Figure 4:**
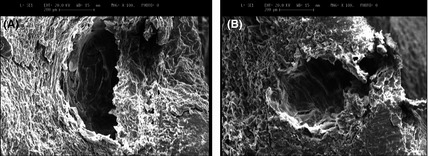
Scanning electron micrographs of *Citrus limon* peels extracted by microwave‐assisted hydrodistillation (MAHD) after 15 min (A) and solvent‐free microwave extraction (SFME) after 15 min (B).

The images from the surfaces of *C. limon* peels obtained by SEM after MAHD and SFME are shown in Figure 4. Both MAHD and SFME resulted in apparent physical changes in the glands of *C. limon* peels after 15 min. While the EO glands of MAHD and SFME were destroyed totally and extractions were done thoroughly, water temperature in HD (72°C) did not reach the boiling point when the extraction had not started yet. These observations confirm that microwave irradiation has a stronger destructive effect on the oil‐bearing structures of *C. limon* peel. Also, microwave irradiation causes the glandular walls to crumble or rupture more rapidly and more efficiently. These results are in good agreement with the findings of Rezvanpanah et al. (2011) in the extraction of EOs from *Satureja hortensis* when using MAHD and HD as extraction methods. They showed that MAHD operates faster in destroying EO‐bearing glands and, as a result, EO diffuses faster into the medium than HD. Lucchesi et al. (2007) extracted EO from cardamom through HD and SFME and presented similar findings. They reported that microwaves seem to cause the rupture of EO glands more rapidly than the conventional HD. In the case of SFME, when the glands were under great thermal stress and when localized high pressures were induced by microwave heating, the pressure build‐up within the glands could have exceeded their capacity for expansion, and hence their rapid rupture compared to the control experiment. This is the mechanism postulated by Chen and Spiro (1995) for the MAE of rosemary leaves.

### Evaluation of physical constants

Table 2 lists the mean values for the physical constants (refractive index, specific gravity, visual appearance, and color) of EOs extracted from *C. limon* peels by HD, MAHD, and SFME. There were no significant differences between the EOs extracted through HD, MAHD, and SFME methods in terms of their specific gravities and refractive indices. However, the visual appearance of the EOs extracted by MAHD and SFME were somewhat brighter than that obtained by HD. A brighter visual appearance in the case of MAHD and SFME was also confirmed by their higher L* and lower b*. Also, specific gravity, refractive index, and visual appearance perceptions of all the samples were within the range indicated by FCC standards (Burdock 2010).

**Table 2:** Physical constants and IC_50_ of essential oils extracted from *Citrus limon* peels by hydrodistillation (HD), microwave‐assisted hydrodistillation (MAHD), and solvent‐free microwave extraction (SFME)

Physical constants	FCC^1^	HD	MAHD	SFME	BHT	Vitamin C
Specific gravity (25^°^C)	0.849–0.855	0.854^a^ 2 ± 0.006	0.827^a^ ± 0.021	0.837^a^ ± 0.012		
Refractive index (20^°^C)	1.473–1.476	1.474^a^ ± 0.001	1.473^a^ ± 0.000	1.473^a^ ± 0.000		
Appearance	Pale yellow	Yellow	Pale yellow	Pale yellow		
L*^3^		63.50^b^ ± 0.60	65.00^ab^ ± 1.20	66.25^a^ ± 1.00		
a*		−11.75^a^ ± 0.50	−8.00^b^ ± 0.00	−10.75^a^ ± 0.50		
b*		10.75^a^ ± 0.50	4.25^c^ ± 0.50	8.75^b^ ± 0.50		
∆E*_ab_ (relative to HD)			7.65	3.54		
IC_50_ (mg/mL)		44.06	42.03	97.23	0.0257	0.0206

BHT, butylated hydroxytoluene. ^1^Standard physical constants of *Citrus limon* essential oils according to Food Chemicals Codex (FCC) (Burdock 2010). ^2^In each row means with different letters are significantly different (*P* < 0.05). ^3^L*: lightness; a*: redness‐greenness; b*: blueness‐yellowness.

Bayramoglu et al. (2008) observed no significant difference between HD and SFME in terms of specific gravities and refractive indices of EOs extracted from oregano. Golmakani and Rezaei (2008a,b) found that specific gravities and refractive indices of thyme EOs extracted by HD and MAHD were similar. Also, they reported that the color of EOs extracted by MAHD was brighter than that of HD. ΔE_ab_ of EOs extracted by MAHD and SFME were 7.65 and 3.54, respectively, relative to the HD as reference, which indicates a clear similarity in the color of EOs extracted by HD and MAE. Therefore, the examination of MAHD and SFME as novel and fast extraction methods do not bear any remarkable standouts in terms of the measured physical constants of the extracted EOs.

### Antioxidant activity: DPPH radical scavenging activity

**Figure 5:**
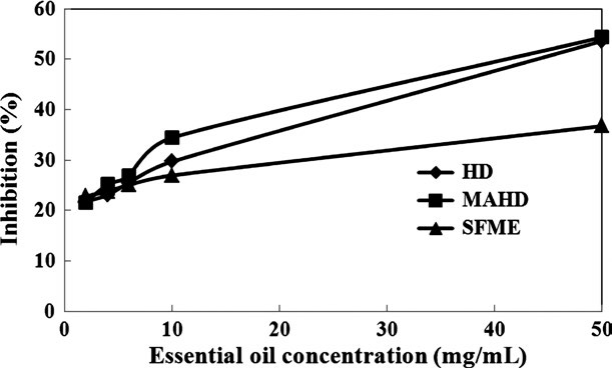
Changes in the inhibition of DPPH radical solutions with different concentrations of essential oils (2, 4, 6, 10, and 50 mg/mL) from *Citrus limon* peel obtained by hydrodistillation (HD), microwave‐assisted hydrodistillation (MAHD), and solvent‐free microwave extraction (SFME).

Figure 5 shows the DPPH radical scavenging activities of different concentrations of EO obtained from *C. limon* peel, extracted via the various extraction methods applied in this study. The radical scavenging activity increased as the concentration of EOs increased. According to Table 2, IC_50_ values of the EOs extracted by HD, MAHD, and SFME were 44.06, 42.03, and 97.23 mg/mL, respectively. In comparison with HD and MAHD, the lower scavenging activity (higher IC_50_ value) of SFME is related to its lower concentrations of antioxidant constituents such as limonene. In contrast to our results, Mazidi et al. (2012) showed that there were no significant differences between the IC_50_ values of Black Zira EOs extracted by Electromantle and the microwave oven. Also, the antioxidant properties relating to the EOs of *C. limon* peels, extracted by HD, MAHD, and SFME were insubstantially compared with that of known synthetic antioxidants, BHT, and vitamin C (Table 2). Furthermore, the IC_50_ of EOs extracted by the three methods measured significantly higher than the IC_50_ of BHT and vitamin C. A lower IC_50_ value reflects a better protective action (Ayoughi et al. 2011). Therefore, radical scavenging activity of *C. limon* EOs was significantly lower than those of BHT and vitamin C.

### GC/MS

Table 3 lists the retention indices, molecular weights, molecular formulas, IUPAC (International Union of Pure and Applied Chemistry) names, CAS (Chemical Abstract Service) and the percentages of areas under the curves of 47 identified constituents, representing about 99% of the total detected constituents obtained from *C. limon* peels by HD, MAHD, and SFME. The constituents are grouped into four classes: monoterpenes, sesquiterpenes, oxygenated terpenes, and alkanes.

**Table 3:** Chemical compositions of essential oils (EOS) obtained from *Citrus limon* peels by HD, MAHD, and SFME using GC/MS

No.	Compounds	IUPAC^1^ Names	CAS Number^2^	Molecular Formula	Molecular Weight (g/mol)	RT^3^ (min)	RI^4^	Relative peak area [%]^5^		
								HD^6^	MAHD^7^	SFME^8^
Oxygenated terpenes										
1	Linalool	3,7‐dimethylocta‐1,6‐dien‐3‐ol	78‐70‐6	C_10_H_18_O	154.25	11.1	1098	0.22^a^	0.13^b^	0.14^b^
2	p‐Cymene	1‐Methyl‐4‐(1‐methylethyl)benzene	99‐87‐6	C_10_H_14_O_2_	134.22	8.5	1024	0.68^a^ *	0.38^b^	0.11^c^
3	cis‐Limonene oxide	4‐Isopropenyl‐1‐methyl‐7‐oxabicyclo[4.1.0] heptan	4680‐24‐4	C_10_H_16_O	152.23	12.5	1131	0.01^a^	0.02^a^	0.01^a^
4	trans‐Limonene oxide	4‐isopropenyl‐1‐methyl‐7‐oxabicyclo[4.1.0] heptan	6909‐30‐4	C_10_H_16_O	152.23	12.6	1135	nd^9^	0.02^a^	0.03^a^
5	Citronellal	3,7‐dimethyloct‐6‐en‐1‐al	106‐23‐0	C_10_H_18_O	154.25	13.2	1150	0.06^b^	0.09^a^	0.08^ab^
6	Terpinene‐4‐ol	4‐methyl‐1‐(propan‐2‐yl)cyclohex‐3‐en‐1‐ol	562‐74‐3	C_10_H_18_O	154.25	14.2	1174	0.31^a^	0.02^b^	0.03^b^
7	α‐terpineol	2‐(4‐Methyl‐1‐cyclohex‐3‐enyl)propan‐2‐ol	98‐55‐5	C_10_H_18_O	154.25	14.8	1187	0.28^a^	0.08^b^	0.08^b^
8	Safranal	2,6,6‐trimethyl‐1,3‐cyclohexadiene‐1‐carboxaldehyde	116‐26‐7	C_10_H_14_O	150.22	15.2	1197	0.03	nd	nd
9	Nerol	(*Z*)‐3,7‐dimethyl‐2,6‐octadien‐1‐ol	106‐25‐2	C_10_H_18_O	154.25	16.3	1225	0.12^b^	0.06^c^	0.22^a^
10	Neral	(2E)‐3,7‐dimethylocta‐2,6‐dienal	5392‐40‐5	C_10_H_16_O	152.23	16.9	1238	0.66^a^	0.30^b^	0.34^b^
11	Carvone	2‐Methyl‐5‐(1‐methylethenyl)‐2‐cyclohexenone	99‐49‐0	C_10_H_14_O	150.22	17.0	1240	0.07^a^	0.02^b^	0.03^b^
12	Geraniol	(*trans*)‐3,7‐dimethyl‐2,6‐octadien‐1‐ol	106‐24‐1	C_10_H_18_O	154.25	17.5	1251	0.06^b^	0.03^b^	0.23^a^
13	Geranial	3,7‐dimethylocta‐2,6‐dienal	5392‐40‐5	C_10_H_16_O	152.23	18.2	1268	0.86^a^	0.38^b^	0.51^b^
14	Perilla aldehyde	(*S*)‐4‐(1‐methylethenyl)‐1‐cyclohexene‐1‐carboxaldehyde	2111‐75‐3	C_10_H_14_O	150.22	18.3	1270	0.03	nd	nd
15	Citronellyl acetate	3,7‐dimethyloct‐6‐en‐1‐yl acetate	150‐84‐5	C_12_H_22_O_2_	198.30	21.6	1350	0.09^b^	0.13^a^	0.16^a^
16	Neryl acetate	(2*Z*)‐3,7‐Dimethyl‐2,6‐octadien‐1‐yl acetate	141‐12‐8	C_12_H_20_O_2_	196.29	22.1	1362	1.16^a^	1.19^a^	1.28^a^
17	Geranyl acetate	3,7‐Dimethyl‐2,6‐octadiene acetate	105‐87‐3	C_12_H_20_O_2_	196.29	22.9	1381	0.51^b^	0.67^a^	0.70^a^
18	α‐Bisabolol	(2R)‐6‐methyl‐2‐[(1R)‐4‐methyl‐1‐cyclohex‐3‐enyl]hept‐5‐en‐2‐ol	23089‐26‐1	C_15_H_26_O	222.37	34.6	1681	nd	nd	0.01
19	n‐Nonanal	Nonanal	124‐19‐6	C_9_H_18_O	142.24	11.3	1102	0.14^a^	0.05^b^	0.04^c^
20	Methyl geranate	methyl 3,7‐dimethylocta‐2,6‐dienoate	1189‐09‐9	C_11_H_18_O_2_	182.26	20.4	1320	nd	nd	0.01
Monoterpenes										
21	Limonene	1‐Methyl‐4‐(1‐methylethenyl)‐cyclohexene	138‐86‐3	C_10_H_16_	152.23	8.9	1036	63.15^a^	61.62^a^	58.58^a^
22	α‐Thujene	1‐isopropyl‐4‐methylbicyclo[3.1.0]hex‐3‐ene	2867‐05‐2	C_10_H_16_	136.24	5.6	924	0.59^a^	0.83^a^	0.82^a^
23	α‐Pinene	(1*S*,5*S*)‐2,6,6‐Trimethylbicyclo[3.1.1]hept‐2‐ene ((−)‐α‐Pinene)	80‐56‐8	C_10_H_16_	136.24	5.8	932	2.64^a^	3.11^a^	3.08^a^
24	Camphene	2,2‐dimethyl‐3‐methylene‐bicyclo[2.2.1]heptane	79‐92‐5	C_10_H_16_	136.24	6.1	946	0.06^b^	0.07^a^	0.07^a^
25	Sabinene	4‐methylene‐1‐(1‐methylethyl)bicyclo[3.1.0]hexane	3387‐41‐5	C_10_H_16_	136.24	6.8	971	1.91^b^	2.54^a^	2.59^a^
26	β‐pinene	6,6‐Dimethyl‐2‐methylenebicyclo[3.1.1]heptane	127‐91‐3	C_10_H_16_	136.24	7.0	977	9.01^a^	8.74^a^	8.56^a^
27	β‐Myrcene	7‐Methyl‐3‐methylene‐1,6‐octadiene	123‐35‐3	C_10_H_16_	136.24	7.3	989	2.66^b^	3.30^a^	3.43^a^
28	α‐phellandrene	2‐Methyl‐5‐(1‐methylethyl)‐1,3‐cyclohexadiene	99‐83‐2	C_10_H_16_	136.24	7.8	1004	0.06^b^	0.09^a^	0.10^a^
29	α‐terpinene	4‐Methyl‐1‐(1‐methylethyl)‐1,3‐cyclohexadiene	99‐86‐5	C_10_H_16_	136.24	8.2	1015	0.33^b^	0.38^ab^	0.43^a^
30	Cis‐β‐Ocimene	(3Z)‐3,7‐Dimethylocta‐1,3,6‐triene	3338‐55‐4	C_10_H_16_	136.24	9.0	1038	0.03^d^	0.05^c^	0.07^a^
31	trans‐β‐Ocimene	(3E)‐3,7‐Dimethylocta‐1,3,6‐triene	3779‐61‐1	C_10_H_16_	136.24	9.3	1046	0.08^c^	0.11^b^	0.18^a^
32	γ‐Terpinene	1‐Methyl‐4‐(propan‐2‐ylidene)cyclohex‐1‐ene	99‐85‐4	C_10_H_16_	136.24	9.7	1059	11.19^a^	12.16^a^	12.71^a^
33	Terpinolene	4‐Isopropylidene‐1‐methylcyclohexene	586‐62‐9	C_10_H_16_	136.24	10.7	1087	0.64^b^	0.81^a^	0.91^a^
Sesquiterpenes										
34	β‐Elemene	*rel*‐(1*S*,2*S*,4*R*)‐1‐methyl‐2,4‐di(prop‐1‐en‐2‐yl)‐1‐vinylcyclohexane	515‐13‐9	C_15_H_24_	204.35	23.3	1388	nd	nd	0.02
35	cis‐α‐Bergamotene	(1S,5S,6S)‐2,6‐Dimethyl‐6‐(4‐methyl‐3‐penten‐1‐yl)bicyclo[3.1.1]hept‐2‐ene	17699‐05‐7	C_15_H_24_	204.35	24.4	1411	0.02^c^	0.05^b^	0.07^a^
36	trans‐α‐Bergamotene	(1S,5S,6R)‐2,6‐Dimethyl‐6‐(4‐methyl‐3‐penten‐1‐yl)bicyclo[3.1.1]hept‐2‐ene	13474‐59‐4	C_15_H_24_	204.35	25.0	1432	0.68^b^	0.78^b^	1.21^a^
37	α‐humulene	2,6,6,9‐Tetramethyl‐1,4‐8‐cycloundecatriene	6753‐98‐6	C_15_H_24_	204.35	25.7	1449	0.01^c^	0.02^b^	0.05^a^
38	(E)‐β‐Farnesene	(6E)‐7,11‐dimethyl‐3‐methylidenedodeca‐1,6,10‐	77129‐48‐7	C_15_H_24_	204.35	25.9	1453	0.02^c^	0.05^b^	0.09^a^
39	Valencene	((2R)‐8,8,8a‐trimethyl‐2‐prop‐1‐en‐2‐yl‐1,2,3,4,6,7‐hexahydronaphthalene	4630‐07‐3	C_15_H_24_	204.35	27.3	1489	0.17^b^	0.12^c^	0.39^a^
40	Bicyclogermacrene	(4E,8E)‐4,8,11,11‐tetramethylbicyclo[8.1.0]undeca‐4,8‐diene	24703‐35‐3	C_15_H_24_	204.35	27.4	1492	0.03^b^	0.03^b^	0.12^a^
41	cis‐α‐Bisabolene	1‐methyl‐4‐[(2Z)‐6‐methylhepta‐2,5‐dien‐2‐yl]cyclohexene	25532‐79‐0	C_15_H_24_	204.35	27.7	1499	0.12^b^	0.11^b^	0.15^a^
42	β‐Bisabolene	(4S)‐1‐Methyl‐4‐(6‐methyl‐1,5‐heptadien‐2‐yl)cyclohexene	495‐62‐5	C_15_H_24_	204.35	27.9	1505	0.98^b^	0.99^b^	1.65^a^
43	(E)‐caryophyllene	(1R,4E,9S)‐4,11,11‐Trimethyl‐8‐methylenebicyclo[7.2.0]undec‐4‐ene	87‐44‐5	C_15_H_24_	204.35	24.3	1415	0.30^b^	0.34^b^	0.57^a^
44	β‐Santalene	(1R,4S,6S)‐6‐Methyl‐5‐methylidene‐6	‐	C_15_H_24_	204.35	26.0	1456	0.01^c^	0.02^b^	0.04^a^
Alkanes										
45	n‐Tridecane	Tridecane	629‐50‐5	C_13_H_28_	184.36	19.4	1296	nd	0.02^a^	0.01^b^
46	n‐Tetradecane	Tetradecane	629‐59‐4	C_14_H_30_	198.39	23.5	1396	0.01^c^	0.04^b^	0.05^a^
47	n‐Pentadecane	Pentadecane	629‐62‐9	C_15_H_32_	212.41	27.6	1496	0.01^b^	0.03^a^	nd
	Oxygenated terpenes							5.29	3.57	4.01
	Monoterpenes							92.35	93.81	91.53
	Sesquiterpenes							2.34	2.51	4.36
	Alkanes							0.02	0.09	0.06

HD, hydrodistillation; MAHD, microwave‐assisted hydrodistillation; SFME, solvent‐free microwave extraction; GC/MS, Gas chromatography/mass spectrometry. * In each row means with different letters are significantly different (*P* < 0.05) for each constituent identified. ^1^International union of pure and applied chemistry chemical. ^2^Abstract Service number. ^3^Retention time. ^4^Retention index (RI) relative to C_5_–C_28_ n‐alkanes on the HP‐5MS column. ^5^Mean ± SD (*n* = 3). ^6^Hydrodistillation. ^7^Microwave‐assisted hydrodistillation. ^8^Solvent‐free microwave extraction. ^9^Not detected.

GC/MS results indicated that the EOs extracted by HD, MAHD, and SFME were quite similar in their composition. Based on the values obtained by GC/MS, it was concluded that there was no significant difference in the quantity of the constituents with relative peak areas more than 2%, with a total percentage of 86.36–88.93% in the different extraction methods. A monoterpene called limonene was the most abundant constituent of the EOs extracted from *C. limon* peels by HD (63.15 ± 4.47), MAHD (61.62 ± 4.36%) and SFME (58.58 ± 4.14%). The EOs also bore γ‐terpinene (11.19–12.71%), β‐pinene (8.56–9.01%), β‐Myrcene (2.66–3.43%), and α‐Pinene (2.64–3.11%), regardless of the three extraction methods. Michaelakis et al. (2009) reported similar findings for the constituents in EOs from Citrus. They showed that limonene was the most abundant constituent in the EOs extracted from the fruit peel of orange (96.2%), lemon (74.3%), and bitter orange (96.7%) when extracted via HD. Also, Ferhat et al. (2006) showed that limonene was the most abundant constituent in the EOs extracted from orange peels, with equivalent relative amounts in both extraction methods: 78.5% for HD and 76.7% for SFME.

EOs extracted by HD showed lower amounts of acetate constituents (1.76%) compared to those isolated by MAHD (1.99%) and SFME (2.14%). This result can be attributed to the fact that the higher extraction time of HD had hydrolytic effects on acetate constituents. Results obtained by GC/MS show in this current study that MAE did not cause the deterioration or loss of volatile components in comparison with HD. Therefore, MAE can be recommended as superior method for the extraction of EO.

### Electric consumption and environmental considerations

**Figure 6:**
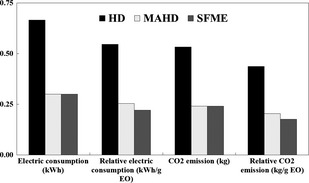
Electric consumption of hydrodistillation (HD), microwave‐assisted hydrodistillation (MAHD), and solvent‐free microwave extraction (SFME) of essential oil (EO) from *Citrus limon* peel.

The reduced extraction time is clearly advantageous for the proposed MAHD and SFME methods in terms of cost and energy. The energy requirement needed to perform the extraction methods, based on the maximum power consumptions of the Electromantle (in HD) and the microwave oven (in both MAHD and SFME), considering the total periods of full extractions, was 0.67 kWh for HD and 0.30 kWh for MAHD and SFME (Fig. 6). Li et al. (2013) showed that for the energy requirements, SFME needs less than 0.5 kWh for normal performance but conventional methods expend more than 4.5 kWh. Relative electric consumption for the production of 1 g EO in HD, MAHD, and SFME was 0.55, 0.25, and 0.22 kWh/g EO, respectively (Fig. 6). This indicates a substantial saving in the extraction cost when using MAHD and SFME instead of HD. Filly et al. (2014) reported that the energy required for the extraction of EOs from rosemary was 4.50 kWh per gram of EO in HD, but 0.25 kWh per gram of EO in SFME.

Regarding the environmental impact of pollution, the calculated quantity of CO_2_ emitted in the atmosphere was higher in the case of HD (0.533 kg CO_2_) than those of MAHD and SFME (0.240 kg CO_2_) (Fig. 6). Golmakani and Rezaei (2008a) found that the amount of CO_2_ which was released into the atmosphere was higher in HD (1600 g CO_2_) than that in MAHD (990 g CO_2_). Relative amounts of CO_2_ emissions that result from the production of 1 g EO were higher in HD (0.437 kg CO_2_/g EO) than those in MAHD (0.203 kg CO_2_/g EO) and SFME (0.176 kg CO_2_/g EO) (Fig. 6). This finding further indicated that there was a significant difference between MAHD and SFME in terms of the amount of CO_2_ released into the atmosphere for the production of 1 g EO. Also, waste water was lower in SFME than HD and MAHD. However, there was a significant difference between MAE and HD in terms of the CO_2_ released into the atmosphere for the production of 1 g EO. Filly et al. (2014) showed that the amount of CO_2_ released into the atmosphere was dramatically higher in HD (3600 g CO_2_/g EO) than that in SFME (200 g CO_2_/g EO). Also, Li et al. (2013) reported that the quantity of CO_2_ calculated to be rejected in the atmosphere is higher in the case of HD (3600 g CO_2_/g EO) than for SFME (200 g CO_2_/g EO).

## Conclusion

The aim of this work was to confirm the efficiency of MAE methods (SFME and MAHD) and to explain how they speed up the extraction process, without causing considerable changes in the EO composition. For this purpose, the experiment's environmentally friendly processes operated rapidly for the extraction of EOs from dry and fresh *C. limon* peels. Both MAHD and SFME resulted in a reduced extraction time compared to the conventional HD technique. No significant differences were found in the physical constants (refractive index, specific gravity, visual appearance, and color) of EOs extracted by HD, MAHD, and SFME. Furthermore, the EOs extracted by the three extraction methods had the same compositions. The antioxidant activities of EOs from *C. limon* peel that had been extracted by HD, MAHD, and SFME were lower than those of BHT and vitamin C. SEM images of *C. limon* peels that had undergone MAHD and SFME indicated that microwave heating causes a quick rupture of EO glands and their surrounding areas, resulting in a shorter extraction process. The amount of CO_2_ emission – a result of the EO extraction process – was dramatically higher in HD than those of MAHD and SFME. Based on our results, MAHD and SFME can be termed as “green” extraction methods (from an energy consumption point of view). In addition to that, MAHD and SFME can also be proposed to be utilized for large‐scale productions of EOs by commercializing the equipment instead of the conventional HD apparatus.

## Conflict of Interest

None declared.
